# Efficacy of Daratumumab-Containing Regimens Among Patients With Multiple Myeloma Progressing on Lenalidomide Maintenance: Retrospective Analysis

**DOI:** 10.3389/fonc.2022.826342

**Published:** 2022-02-18

**Authors:** Hira Mian, Christine Eisfeld, Christopher P. Venner, Esther Masih-Khan, Moustafa Kardjadj, Victor H. Jimenez-Zepeda, Cyrus Khandanpour, Georg Lenz, Arleigh McCurdy, Michael Sebag, Kevin Song, Richard LeBlanc, Darrell White, Julie Stakiw, Anthony Reiman, Martha Louzada, Muhammad Aslam, Rami Kotb, Engin Gul, Donna Reece

**Affiliations:** ^1^ Department of Oncology, Juravinski Cancer Center, Hamilton, ON, Canada; ^2^ Department of Medicine, University Hospital Münster, Münster, Germany; ^3^ Cross Cancer Institute, University of Alberta, Edmonton, AB, Canada; ^4^ Department of Medical Oncology and Hematology, Princess Margaret Cancer Centre, Toronto, ON, Canada; ^5^ Canadian Myeloma Research Group, Toronto, ON, Canada; ^6^ Arnie Charbonneau Cancer Institute, University of Calgary, Calgary, AB, Canada; ^7^ Department of Medicine, The Ottawa Hospital, Ottawa, ON, Canada; ^8^ Department of Medicine, McGill University, Montreal, QC, Canada; ^9^ BC Cancer Agency, Vancouver General Hospital, Vancouver, BC, Canada; ^10^ Maisonneuve-Rosemont Hospital Research Centre, University of Montreal, Montreal, QC, Canada; ^11^ Queen Elizabeth II Health Sciences Centre, Dalhousie University, Halifax, NS, Canada; ^12^ Saskatoon Cancer Centre, University of Saskatchewan, Saskatoon, SK, Canada; ^13^ Department of Medicine, Saint John Regional Hospital, Saint John, NB, Canada; ^14^ Department of Haematology, London Regional Cancer Center, London, ON, Canada; ^15^ Department of Medical Oncology, Allan Blair Cancer Centre, Regina, SK, Canada; ^16^ Department of Medical Oncology & Hematology, Cancer Care Manitoba, Winnipeg, MB, Canada

**Keywords:** multiple myeloma, lenalidomide, maintenance, daratumumab (DARA), relapsed/refractory

## Abstract

**Background:**

Daratumumab, a monoclonal antibody directed against CD38 is a recent class of drugs introduced into the multiple myeloma therapeutic landscape. While clinical trial data have shown a remarkable impact on outcomes, the efficacy of daratumumab combination therapies in specific clinically relevant subgroups including among patients refractory to lenalidomide maintenance remains unknown.

**Methods:**

In this study, retrospective data were reviewed from the Canadian Myeloma Research Group and the German Munster Myeloma databases to identify patients that received daratumumab in combination with pomalidomide (DPd), lenalidomide (DRd), and bortezomib (DVd) in a population that had relapsed on lenalidomide maintenance postautologous stem cell transplant. The primary aim of the study was to look at outcomes of these patients in different daratumumab combinations.

**Results:**

A total of 73 patients were identified. The median age of the patients at the time of daratumumab initiation was 60 (38-72) and 64.4% (*n* = 47) were men. In the selected cohort, 43.8% (*n* = 32) were treated with DRd, 31.5% (*n* = 23) with DVd, and 24.7% (*n* = 18) with DPd regimen. The median progression-free survival (PFS) of the entire cohort was 15.8 months (95% CI, 12.9–37.1 months). The median PFS of the individual regimens was as follows: DPd 18.9 months (95% CI, 13.7-not reached), DRd 21.7 months (95% CI, 11.6-not reached), and DVd 12.9 months (95% CI, 3.1-not reached).

**Conclusions:**

Daratumumab-containing therapies are effective regimens in patients progressing on lenalidomide maintenance. Additional studies are required to decide the optimal regimen post-lenalidomide maintenance.

## Introduction

Multiple myeloma is an incurable plasma cell neoplasm characterized by the clonal proliferation of malignant plasma cells within the bone marrow (1). The clinical manifestations of multiple myeloma, which reflect end organ damage, include renal impairment, hypercalcemia, lytic bony lesions, and anemia. Treatment modalities for multiple myeloma have led to pivotal improvements in patient outcomes in the past decades with many new therapeutic agents entering the landscape (2).

Lenalidomide maintenance following autologous stem cell transplant (ASCT) remains a standard of care among transplant-eligible patients with newly diagnosed multiple myeloma (NDMM) (3). The monoclonal antibody, daratumumab, represents a novel class of drugs that has shown remarkable efficacy among both newly diagnosed and relapsed patients in landmark clinical trials (4, 5). Daratumumab-containing triplet regimens have been introduced for patients in the relapsed setting, including in combination with dexamethasone and pomalidomide (DPd), lenalidomide (DRd), or bortezomib (DVd). The efficacy of these daratumumab-containing regimens in patients progressing on low-dose maintenance lenalidomide following the first line of therapy is largely undescribed. Landmark randomized clinical trials such as APOLLO, POLLUX, or CASTOR have either excluded or included only a very small proportion of patients progressing specifically on lenalidomide maintenance (4, 6, 7). To our knowledge, there is no prospective data to allow comparison of the efficacy of these three regimens in patients specifically progressing on lenalidomide maintenance. This is an increasingly common clinical scenario, and understanding outcomes and gaps of various combination regimens can further improve clinical decision-making.

In order to fulfill this knowledge gap, we report on the outcomes of DPd, DRd, and DVd regimens given as a second-line therapy for patients progressing specifically on lenalidomide maintenance following frontline ASCTs. Using two large disease-specific databases, we aimed to understand the response rates, progression-free survival, and overall survival in patients treated with DPd, DRd, and DVd following progression on lenalidomide maintenance.

## Materials and Methods

### Data Source

The Canadian Myeloma Research Group Database is a prospectively maintained disease-specific database with over 7,000 patients enrolled from 14 academic sites across Canada with legacy data collected from 2007. The Munster Myeloma database collects myeloma-specific information in a German academic center and currently contains data from 800 patients from 2005. All patients treated with daratumumab-based regimens in second line, including those treated on clinical trial protocols, following relapse on lenalidomide maintenance were included in the analysis from the two databases analyzed up to June 30, 2020. Local research ethics boards at every contributing site approve entry of source data into the CMRG-DB. The approval for review of this specific dataset was obtained from the University Health Network Research Ethics Board (UHN REB) as per the approved governance structure of the CMRG-DB, and the analysis was conducted in accordance with the Declaration of Helsinki.

### Included Patient Cohort

Multiple myeloma patients progressing on or within 60 days of last receiving lenalidomide-based maintenance therapy after high-dose chemotherapy and ASCT who were then treated with DPd, DRd, or DVd between Jan 2015 and Jun 2020 were identified using the Canadian Myeloma Research Group Database and the Munster Myeloma database. As this was a retrospective study, patients treated on clinical trials were also included in our cohort.

### Study Outcomes

The primary endpoint of this study was progression-free survival (PFS). PFS was defined from the date of daratumumab-based regimen initiation until the date of progression or death, whichever came first. Secondary endpoints included response (response assessed as per standard International Myeloma Working Group Response Criteria) (8) and overall survival (OS). OS was calculated from the date of daratumumab-based regimen initiation to date of death or censored at date of last follow-up.

### Statistical Plan

Patient-baselined demographics, disease characteristics, and treatment details (induction therapy, maintenance therapy, and daratumumab-based regimens) were analyzed using descriptive statistics. Categorical variables and continuous variables were analyzed using Fisher’s exact test and the Wilcoxon ran-sum test, respectively. The Kaplan-Meier method and log rank test was used to estimate the time-to-event endpoints and between group comparisons for PFS and OS. Statistical analyses were performed using R (4.1.0) and RStudio (1.4.1717) for Windows. All *p*-values were two sided; *p* < 0.05 was considered to indicate a statistically significant result.

## Results

A total of 1,380 NDMM patients who received lenalidomide maintenance were identified in the two databases. Of those, 73 patients were treated with a daratumumab-containing regimen on progression in second line. In the included cohort, 32 patients (43.8%) were treated with DRd, 23 (31.5%) with DVd, and 18 (24.7%) with DPd regimen. The baseline characteristics for the entire cohort as well as for each group (DPd, DRd, DVd) are summarized in **Table 1**. The median age for the entire cohort was 60 years and 47 (64.4%) were male. The most common myeloma subtype was IgG in 40 (54.8%) of the patients. The majority of patients were ISS stage II at diagnosis 47.1% (*n* = 32). High-risk status based on the presence or absence of t(4:14) or t(14:16) or deletion17p was available in 86% (*n* = 63) of patients of which 17.5% were high risk. Most patients on DPd had their therapy initiated in 2017 (88.9%), whereas those on DRd (75.0%) and DVd (60.9%) initiated it most commonly in 2019. 

**Table 1:** Patient characteristics at diagnosis for the daratumumab combination treatment groups.

Characteristics	All (*N* = 73)	DPd (*N* = 18)	DRd (*N* = 32)	DVd (*N* = 23)
**Age at initiation of daratumumab [median (range)]**	60 (38–72)	53 (38–68)	59 (39–71)	64 (47–72)
**Male [*n* (%)]**	47 (64.4)	15 (83.3)	19 (59.4)	13 (56.5)
**MM subtype [*n* (%)]**				
**IgG**	40 (54.8)	14 (77.7)	14 (43.8)	12 (52.2)
**IgA**	17 (23.3)	3 (16.7)	7 (21.9)	7 (30.4)
**FLC**	14 (19.2)	1 (5.6)	10 (31.2)	3 (13.1)
**Other**	2 (2.7)	0	1 (3.1)	1 (4.3)
**ISS stage at diagnosis [*n* (%)]**				
**I**	23 (33.8)	4 (23.5)	11 (36.7)	8 (38.1)
**II**	32 (47.1)	10 (58.8)	12 (40.0)	10 (47.6)
**III**	13 (19.1)	3 (17.7)	7 (23.3)	3 (14.3)
**Unknown**	5	1	2	2
**High-risk FISH** **^a^** **[*n* (%)]^b^**				
**Present**	11 (17.5)	2 (12.5)	4 (14.8)	5 (25.0)
**Not present**	52 (82.5)	14 (87.5)	23 (85.2)	15 (75.0)
**Unknown**	10	2	5	3
**Initiation year [*n* (%)]**				
**2015–2016**	2 (2.7)	1 (5.6)	1 (3.1)	0
**2017**	17 (23.3)	16 (88.9)	1 (3.1)	0
**2018**	9 (12.3)	1 (5.6)	5 (15.6)	3 (13.0)
**2019**	38 (52.1)	0	24 (75.0)	14 (60.9)
**2020**	7 (9.6)	0	1 (3.1)	6 (26.1)

a High-risk cytogenetics defined as del 17p, t(4;14), and/or t(14;16).

^b^Denominator for percentage calculations do not include unknown values.

ISS, International Staging System; CR, complete response; VGPR, very good partial response; PR, partial response; MR, minimal response; SD, stable disease; PD, progressive disease; Len, lenalidomide.

Regarding the individual subgroups, the median age for the group receiving DPd and with the most clinical trial patients (94%, *n* = 17) was 53 years (range 38–68), younger than the other two groups. IgG MM subtype was the most common in all the three subgroups. The ISS stage was well balanced between the three groups. There was a slightly higher proportion of patients with high-risk cytogenetics in the DVd arm; however, the overall number of patients were small in each cytogenetic risk group.

Regarding outcomes of first-line therapy preceding a daratumumab-based regimen, the details are outlined in **Table 2**. Most patients (>80%) received CyBorD as induction and had a greater than 90% overall response on it in the entire cohort. Three patients received tandem ASCTs, 1 in the DPd group and 2 in the DRd groups. Post-ASCT, all patients received lenalidomide maintenance as a single agent. Patients received single-agent lenalidomide doses between 5 and 15 mg, 48% (*n* = 35) on a 21- of 28-day-cycle schedule and others (52% *n* = 38) on a continuous 28 of 28-day-cycle schedule. All patients progressed while on maintenance; however, the most frequent maintenance dose was 10 mg (90.1%) at the time of progression. Maintenance median duration was 21.1 months (range, 1.0–77.6 months) for the entire cohort. Among the specific subgroups, the median duration on lenalidomide maintenance was 23.9 months (range, 3.1–77.2) for the DPd group, 19.0 months (range, 1.4–67.7) for the DRd group, and 16.4 months (range, 1.0–77.6) for the DVd group. For the entire cohort, the median PFS on first-line treatment was 33.8 months (95% CI, 30.5–37.5).

**Table 2:** Summary of frontline therapy for the daratumumab combination treatment groups.

Frontline therapies	All (*N* = 73)	DPd (*N* = 18)	DRd (*N* = 32)	DVd (*N* = 23)
**Induction therapy**				
**Regimen [*n* (%)]**				
**CyBoRD**	61 (83.6)	16 (89)	26 (81.3)	19 (82.6)
**VD**	4 (5.5)	1 (6)	1 (3.1)	2 (8.7)
**RVD**	2 (2.7)	1 (6)	1 (3.1)	0
**Other**	6 (8.2)	0	4 (12.5)	2 (8.7)
**Best response on induction [*n* (%)]**				
**ORR (>PR)**	67 (94.4)	16 (94.1)	30 (93.8)	21 (95.4)
**CR/VGPR**	41 (57.8)	5 (29.4)	24 (75.0)	12 (54.5)
**PR**	26 (36.6)	11 (64.7)	6 (18.8)	9 (40.9)
**MR/SD**	4 (5.6)	1 (5.9)	2 (6.2)	1 (4.5)
**Unknown**	2	1	0	1
**Maintenance therapy**				
**Len maintenance dose at progression [*n* (%)]**				
**5 mg**	4 (5.6)	0	4 (12.9)	0
**10 mg**	64 (90.1)	17 (94.4)	25 (80.6)	22 (100.0)
**15 mg**	3 (4.3)	1 (5.6)	2 (6.5)	0
**Unknown**	2	0	1	1
**Tandem ASCT**	3	1	2	0
**Len maintenance duration (months; median, range)**	21.1 (1.0–77.6)	23.9 (3.1–77.2)	19.0 (1.4–67.7)	16.4 (1.0–77.6)
**Median PFS for first-line treatment (months, 95% CI)**	33.8 (30.5–37.5)	35.6 (31.9–56.5)	32.5 (26.9–55.4)	31.1 (23.0–38.2)

Denominator for percentage calculations do not include unknown values.

Cy, cyclophosphamide; Bor, bortezomob; V, velcade; D, dexamethasone; Len, lenalidomide; CR, complete response; VGPR, very good partial response; PR, partial response; MR, minimal response; SD, stable disease; PD, progressive disease; PFS, progression-free survival; CI, confidence interval.

**Figure 1:**
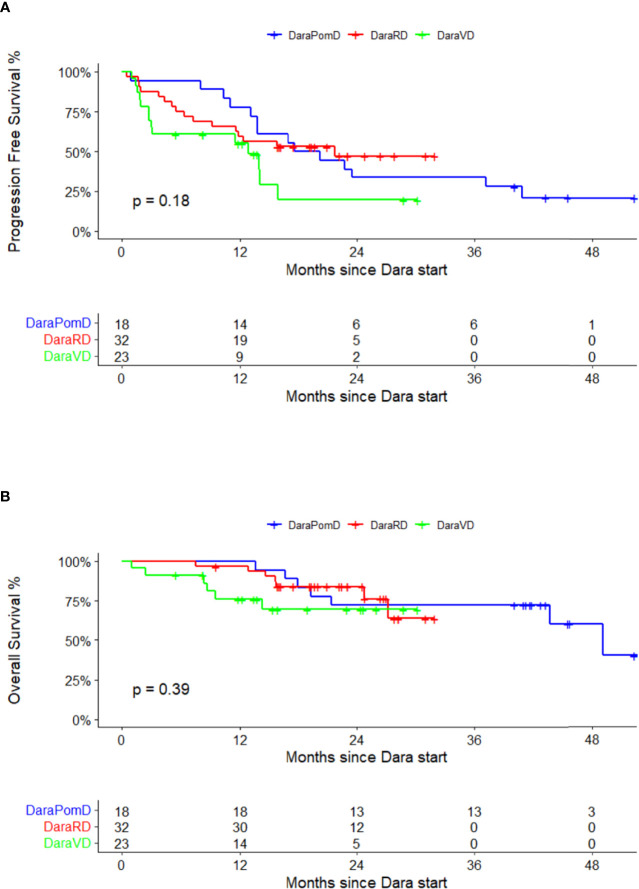
Daratumumab-containing regimens post-lenalidomide maintenance. **(A)** Progression-free survival. **(B)** Overall survival.

Baseline lab values at initiation of daratumumab are summarized in **Table 2**. The median follow-up for the entire cohort from the time of daratumumab initiation was 21.0 months (range, 0.9–30.2). The median follow-up for DPd, DRd, and DVd regimen was 41.8 months (range, 13.6–53.6 months), 21.6 months (range, 7.5–32.0 months) and 13.8 months (range, 0.9–30.2 months), respectively. The response of each daratumumab-containing regimen is outlined in **Table 3**. A higher proportion of patients in the DPd arm (76.5%) obtained a CR/VGPR compared with DRd (58.1%) or DVd (28.6%). The median PFS of the entire cohort was 15.8 months (95% CI, 12.9–37.1 months). The median PFS of the individual regimens was as follows: DPd 18.9 months (95% CI, 13.7-not reached), DRd 21.7 months (95% CI, 11.6-not reached), and DVd 12.9 months (95% CI, 3.1-not reached) as demonstrated in **Figure 1A** (*p*-value = 0.18). The median OS for the entire cohort was 49.1 months (95% CI, 43.7-not reached). The median OS for DPd was 49.1 months (2-year OS, 72.2%) and was not reached for DRd (2-year OS, 84.0%) or DVd (2-year OS, 69.2%) as shown in **Figure 1B**.

**Table 3:** Baseline lab values at initiation and responses on daratumumab based therapy.

Daratumumab combination therapy	All (*N* = 73)	DPd (*N* = 18)	DRd (*N* = 32)	DVd (*N* = 23)
**Lab values at initiation of daratumumab therapy**				
**Hemoglobin [g/L, median (range)]**	120 (72–164)	128 (87–154)	120 (82–162)	120 (72–164)
**White blood cell count ×10^9^/L [median (range)]**	4.0 (1.9–15.5)	4.4 (2.5–15.5)	4.1 (1.4–10.9)	3.3 (1.9–6.9)
**Absolute neutrophil count ×10^9^/L [median (range)]**	2.0 (0.5–10.2)	2.0 (1.2–10.2)	2.2 (0.5–6.9)	1.8 (0.9–5.4)
**Platelet count ×109/L [median (range)]**	143 (16–371)	159 (32–275)	149 (42–371)	127 (16–218)
**Calcium [mg/dl, median (range)]**	2.0 (1.9–3.4)	2.3 (2.1–2.4)	2.4 (2.0–2.7)	2.3 (2.0–3.4)
**Creatinine [µmol/L, median (range)]**	87 (54–620)	96 (58–136)	85 (58–225)	86 (54–620)
**Albumin [g/L, median (range)]**	38 (19–46)	38 (30–42)	40 (22–46)	36 (19–44)
**LDH [U/L, median (range)]**	167 (93–861)	179 (102–861)	181 (115–262)	156 (93–449)
**Patients treated on a clinical trial**	21 (28.8)	17 (94.0)	3 (9.4)	1 (4.5)
**Best response to second-line treatment [*n* (%)]**				
**CR/VGPR**	37 (53.6)	13 (76.5)	18 (58.1)	6 (28.6)
**PR**	16 (23.2)	2 (11.8)	6 (19.4)	8 (38.1)
**MR/SD**	8 (11.6)	1 (5.8)	2 (6.5)	5(23.8)
**PD**	8 (11.6)	1 (5.8)	5 (16.1)	2 (9.5)
**Unknown**	4	1	1	2

LDH, lactate dehydrogenase; CR, complete response; VGPR, very good partial response; PR, partial response; MR, minimal response; SD, stable disease; PD, progressive disease; PFS, progression-free survival; CI, confidence interval.

## Discussion

Our study compares the outcomes of DPd, DRd, and DVd regimens in patients progressing on lenalidomide maintenance. The results from this study provide a benchmark for outcomes expected with these regimens in this specific clinical setting and highlights opportunities for further improvement.

Daratumumab-containing regimens are shown to be effective post-lenalidomide maintenance (9); however, the efficacy of specific combinations remains unknown. The results presented here are in line with recent subanalyses of studies examining patients progressing on lenalidomide following one prior line of treatment. In the phase II nonrandomized MM-014 trial, DPd was evaluated in patients following one to two prior lines of treatment. DPd was associated with a median PFS of 21.8 months among those with lenalidomide refractoriness (10) in keeping with our results (median PFS of 18.9 months). DPd was also evaluated in the phase III APOLLO trial in patients who had received one or more prior lines of treatment including lenalidomide and a proteasome inhibitor and demonstrated a median PFS of 12.6 months (6). Although 62% of patients included in the APOLLO study were refractory to lenalidomide as the last previous line of therapy, only 11% of them had received one prior line of therapy, limiting our ability to understand the efficacy of this regimen specifically among patients refractory to lenalidomide maintenance given at first line.

The pivotal trial POLLUX evaluated DRd among patients with one to three prior lines of treatment but excluded patients with lenalidomide refractory disease (4). Kunacheewa et al. evaluated the outcomes of lenalidomide retreatment with triplet regimens among 64 patients progressing on lenalidomide maintenance (11). In this study, ORR was 58% and median PFS was 20.2 months among patients treated with novel triplets following one line of treatment; however, only eight patients were treated with DRd following lenalidomide maintenance (11). Lastly, in the phase III CASTOR study, DVd had a median PFS of 7.8 months among the 60 patients refractory to lenalidomide (12). However, the included patients in the CASTOR study were heterogeneous with more than one prior line of treatment as compared with our study that included patients progressing on lenalidomide maintenance in first line and showed a median PFS of 12.9 months.

Our results demonstrated that daratumumab-based regimens remain effective in patients progressing on lenalidomide maintenance. DPd was effective providing a median PFS of 18.9 months; however, additional data on patients treated off clinical trial are needed to understand the efficacy of this regimen in the “real world”. Moreover, our study also examines the specific use of DRd after progression on lenalidomide maintenance therapy with the results demonstrating that the increased dose of lenalidomide along with the addition of daratumumab can still lead to clinical meaningful disease control in a subset of patients. Additionally, this response appeared to be at least as favorable as DVd, which is commonly used and reimbursed regimen for the treatment of lenalidomide refractory patients based upon the CASTOR registrational trial.

The strength of our study is robust information on myeloma-specific variables on patients’ progressing on lenalidomide maintenance, a subgroup with a paucity of data in the literature. The limitation of our study is that while our study consists of real-world patients, patients on clinical trials were also included. Clinical trial patients may have different outcomes compared to patients not eligible for clinical trials (13); however, further subgroup analysis based upon additional factors including trial participation, cytogenetic risk, and response to first-line treatment could not be conducted in our study due to the sample size. Furthermore, the exact reason why one regimen was picked over another available regimen cannot be elucidated from our study. Given the retrospective collection of this data, toxicities including infection rates were not collected precluding our ability to comment on the safety profile of each regimen. Lastly, our study does not contain information on other emerging daratumumab-containing regimens, such as in combination with carfilzomib (DKd) (14).

In conclusion, our study shows the effectiveness of daratumumab-containing regimens among patients refractory to lenalidomide maintenance following first line ASCT. Additional studies with longer follow up are required to assess the optimal daratumumab based regimen use in this growing population of patients relapsing after lenalidomide maintenance.

## Data Availability Statement

The datasets presented in this article are not readily available because Research Ethics Boards at data contributing sites do not allow patient level data to be shared. Any aggregate data supporting the findings of this study can be made available from the corresponding author upon reasonable request. Requests to access the datasets should be directed to esther.masih-khan@uhnresearch.ca.

## Ethics Statement

The studies involving human participants were reviewed and approved by University Health Network Research Ethics Board. Written informed consent for participation was not required for this study in accordance with the national legislation and the institutional requirements.

## Author Contributions

HM, CE, CV, EM-K, and DR designed the research, performed the research, collected, analyzed, and interpreted data, and wrote the manuscript. MK performed statistical analysis and interpreted data. All authors contributed to data collection, interpreted the data, and reviewed the manuscript.

## Funding

This work was supported by funds from the Canadian Myeloma Research Group.

## Conflict of Interest

HM: Honoraria: Celgene, Janssen, Amgen, Takeda, Sanofi, and GSK. Awards: Research Early Career Award from Hamilton Health Sciences Foundation. Research funding: Janssen. CV: Honoraria: Janssen, Amgen, and Takeda. Research funding: Celgene and Amgen. VJ-Z: Honoraria: BMS, Amgen, Takeda, and Janssen. AM: Honoraria: Celgene, Janssen, Amgen, Takeda, Sanofi, and GSK. MS: membership on an entity’s Board of Directors or advisory committees: Janssen Inc., Amgen Canada, Takeda Canada, and Celgene Canada. KS: Research funding: Celgene. Honoraria: Celgene, Janssen, Amgen, and Takeda. RL: Membership on an entity’s Board of Directors or advisory committees: Celgene; Canada; Janssen Inc.; Amgen Canada; Takeda Canada; Research Funding: Celgene; White: Honoraria and consultancy: Amgen, Celgene, Janssen, Sanofi, Takeda; Louzada: Honoraria: Janssen, Celgene, Amgen, Pfizer; Kotb: Research funding: Merck, Sanofi. Ownership/Share holder: Karyopharm. Honoraria: Celgene/BMS, Janssen, Takeda, Amgen, Sanofi, Merck; Reece: Research funding: Otsuka, Celgene, Janssen, Takeda, Merck, BMS, and Millennium. Consultancy: Celgene, Jansen, Amgen, Karyopharm, and Takeda. Honoraria: Celgene, Janssen, Amgen, Takeda, and GSK.

The remaining authors declare that the research was conducted in the absence of any commercial or financial relationships that could be construed as a potential conflict of interest.

## Publisher’s Note

All claims expressed in this article are solely those of the authors and do not necessarily represent those of their affiliated organizations, or those of the publisher, the editors and the reviewers. Any product that may be evaluated in this article, or claim that may be made by its manufacturer, is not guaranteed or endorsed by the publisher.
